# Automatic hammering of nano-patterns on special polymer film by using a vibrating AFM tip

**DOI:** 10.1186/1556-276X-7-456

**Published:** 2012-08-13

**Authors:** Xiaodong Hong, Yongkang Yang, You Wang

**Affiliations:** 1 College of Materials Science and Engineering, Liaoning Technical University, Fuxin, 123000, China; 2 Materials Physics and Chemistry Department, Harbin Institute of Technology, Harbin, 150001, China; 3 Key Laboratory of Micro-Systems and Micro-Structures Manufacturing, Ministry of Education, Harbin, 150001, China

**Keywords:** Automatic nanolithography, Atomic force microscopy, Poly(styrene-ethylene/butylenes-styrene)

## Abstract

Complicated nano-patterns with linewidth less than 18 nm can be automatically hammered by using atomic force microscopy (AFM) tip in tapping mode with high speed. In this study, the special sample was thin poly(styrene-ethylene/butylenes-styrene) (SEBS) block copolymer film with hexagonal spherical microstructures. An ordinary silicon tip was used as a nano-hammer, and the entire hammering process is controlled by a computer program. Experimental results demonstrate that such structure-tailored thin films enable AFM tip hammering to be performed on their surfaces. Both imprinted and embossed nano-patterns can be generated by using a vibrating tip with a larger tapping load and by using a predefined program to control the route of tip movement as it passes over the sample’s surface. Specific details for the fabrication of structure-tailored SEBS film and the theory for auto-hammering patterns were presented in detail.

## Background

Nano-sized patterns can be created on sample surface using a variety of different surface modification techniques. As a complement to conventional photo and electron-beam lithography, atomic force microscopy (AFM) nanolithography appears to be a unique tool with nanometer accuracy. High resolution AFM nanolithography also provides the ability to create various specialized site-specific nano-patterns or localized functional surface structures; with the integration of additional measurement modules, it allows for the physical and morphological qualities of surface undulations to be immediately characterized. This combined fabrication and characterization function in AFM nanolithography allows convenient *in situ* and in-line pattern creation and characterization [1-6].

There are many AFM nanolithography techniques for surface modification. Some techniques rely on mechanical (contact) interactions between the probe and the sample [6-9]. Various hard or soft materials can be directly removed by the typical mechanical [9,10] or thermomechanical [11] scratching technique. In this field, soft polymer materials are widely used as masking or resisting materials in the fabrication of many devices, and polymer films have been also used to create patterns in their surfaces by an AFM tip with a radius of about 10 nm [12,13]. Sohn and Willett [14] described a novel technique for plowing patterns in masks or resistance material on an arbitrary substrate, in which material can then be deposited for the fabrication of metallic nanostructures. Cappella and Sturm presented a dynamic plowing nanolithography method (DPL) [15-17]. In their method, surface modification occurred through plastic deformation of the material surface after being plowed by a vibrating tip. They also compared DPL with indentation by means of force displacement curves; results showed that the border walls surrounding the lithographed structures using DPL method were much bigger than those created through indentation. Another branch of AFM nanolithography involves the flow of electrical current between the probe (or tip) and the sample. In this field, local oxidation of silicon surfaces is used as a very promising lithographic method by which to create a modified surface at a nanometer scale. Garcia et al. [6] studied the effects and results for the reproducibility, voltage dependence, and kinetics for when oxidation occurred by using dynamic force microscopy modes. In another study involving electrical current and oxidation, Perez-Murano et al. [7] adopted a local oxidation method to grow silicon oxide mounds with nanometer scale on the Si(100) surfaces exposed to air by utilizing an AFM tip in tapping mode. While Gan et al. [8] proposed automatic patterning with AFM through local anodic oxidation, with numerous oxide structures being successfully created to demonstrate the ability of this low cost and accurate system.

In the past studies, we have demonstrated that poly(styrene-ethylene/butylenes-styrene) (SEBS) monolayer thin films which possess a well-ordered hexagonal spheres nanostructure [18] are suitable for hammering nano-patterns in their surfaces by using vibrating AFM tip manually. In the tailored SEBS films, hexagonal-spherical polystyrene (PS) plastic microdomains are embedded in poly(ethylene/butylenes) (PEB) rubber matrix. When the PS microdomain arrays are selectively indented by AFM tip hamming under hard tapping conditions, either imprinted or embossed patterns can be generated. The complete process is documented in our paper titled ‘AFM tip hammering nanolithography’ (ATHN) [19]. However, manual AFM tip hammering lithography has some rough qualities and restrictive limitations: it is a slow, tedious and complicated nanolithography method and prone to error, which limits its promise in potential applications. Based on our studies in ATHN, an improved AFM tip hammering nanolithography method is proposed in this article. The method improves upon the capabilities for AFM tip hammering via a different process, which results in improving the hammering speed and precision greatly. Complicated nano-patterns can now be hammered out by using automatic methods in accordance with the preprogrammed routes. The system allows for high-speed, high resolution, and exact duplication in the hammering of various complicated nano-patterns on tailored SEBS film surfaces. The specifics for the method, the process and examples are presented in the following work.

## Methods

### Materials

Poly(styrene-ethylene/butylenes styrene) triblock copolymer, coded as Kraton G1650, manufactured by Shell Co. (Shell Oil Co., Houston, TX, USA), with PS’s molecular weight of 10,300 g/mol and PEB’s of 53,300 g/mol, polydispersity of 1.19, and polystyrene content of 29 wt.%. Xylene and cyclohexane were used as supplied from TEDIA Co. Ltd. (Fairfield, OH, USA).

### Preparation of sample films

The powder of SEBS G1650 was dissolved in xylene to form a solution with concentration of 0.3 wt.%. After drop-casing the solution onto freshly cleaved mica surface, monolayer SEBS films of 35 nm thick were prepared. The film thickness was measured using an AFM tip scratch technique that we previously developed, and the detailed description of the measurement procedure is given elsewhere [20].

### Solvent annealing procedure

Solvent annealing treatments were made at a temperature of 20°C ±1°C as detailed below. A piece of filter paper was put into a Petri dish to cover its bottom. The mica substrates with the sample films on top were put on the filter paper. After adding 60 μl cyclohexane on the filter paper surrounding the samples, the dish was closely covered by putting a 500 g of dead weight on top. The filter paper is always kept wet by adding additional cyclohexane when necessary according to the judgment of naked eyes in the whole solvent vapor annealing process. The sample was annealed for about 40 h.

### Equipment and software

A MultiMode NanoScope IIIa SPM (Veeco Instruments, Plainview, NY, USA) and a commercial probe NSG 10 (NT-MDT Company, Zelenograd, Moscow, Russia) with a force constant of 5.5 to 22.5 N/m were used, and resonant frequency of tip was around 255 kHz. Nanolithography software version 5.12 [21] was used for automatically controlling the predefined routes in the hammering of the nano-patterns, and Microsoft Visual C^++^ Compiler (Microsoft Corporation, Shanghai, China) was needed to write the program.

## Results and discussion

**Figure 1:**
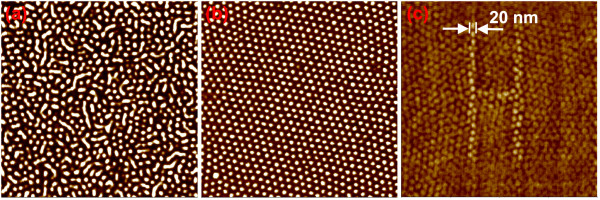
**AFM phase images of thin SEBS films.** (**a**) as-cast film; (**b**) after annealing in cyclohexane vapor for 41 h; (**c**) embossed letter H pattern made by manual ATHN. Image size: 1.0 × 1.0 μm^2^.

Shown in Figure 1a is AFM phase image of the as-cast morphology of a 35-nm thick SEBS G1650 thin film prepared from solution in xylene. The brighter domains in AFM phase images correspond to PS phase. It can be seen from Figure 1b that after being annealed in cyclohexane vapor for about 40 h, the poorly ordered PS cylinders have transformed into well-ordered hexagonal PS spheres. Using structure-tailored monolayer SEBS thin film as media, ATHN [19] was developed. An oscillating AFM tip in tapping mode was used as a nano-hammer forging the special sample surface to ‘write’ either imprinted or embossed patterns with a sub-20-nm linewidth resolution. Figure 1c shows an embossed letter ‘H’ pattern made up of a single array of PS spheres with a diameter of 20 nm, which was generated by ATHN using manual procedure.

**Figure 2:**
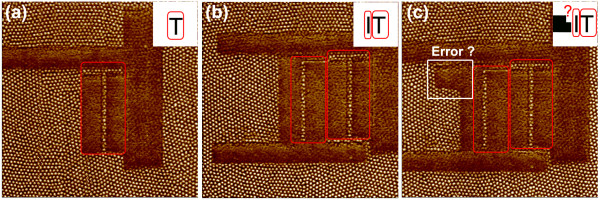
**Processes for hammering of embossed patterns by manual ATHN.** (**a**) fabricating letter T, (**b**) fabricating letter I, and (**c**) making an error in the process of fabricating letter H and failing to generate the entire pattern of HIT. Image size: 2.0 × 2.0 μm^2^.

In the process of manual ATHN, complicated patterns were composed by hammering individual lines or areas one by one to eventually form the desired overall pattern image. In order to accomplish the complicated nano-pattern successfully, detailed planning was needed to design the line or area sizes, the junction point location, and the hammering sequence. Figure 2a-c shows the process for generating a complicated embossed pattern-word ‘HIT’ by manual ATHN. Figure 2a shows the first step involved in creating an embossed letter ‘T’, through the hammering out of four different surrounding areas. Figure 2b shows the second character ‘I’ being generated in the same way. Figure 2c shows an obvious fault in the attempted hammering of the letter ‘H’ character. The error means the failure of manual writing the embossed pattern-word HIT. The failed hammering process highlights obvious shortcomings and drawbacks with ATHN manual procedure, such as complicated operation, low efficiency, and error-prone.

In order to overcome these problems, an improved automatic system for the hammering of complex nano-patterns is proposed on the basis of Nanolithography Software 5.12 [21]. Next, nanolithography software was introduced simply. The nanolithography feature allows for the performing tip moving in nanometer accuracy. Lithography programs direct the microscope for inscribing or moving items on a sample surface. Nanolithography is achieved by using a tip as a hammering tool to create nano-patterns on the sample surface. Microsoft C programming language, along with NanoScript^TM^™ macro litho functions, is used to manipulate the tip-to-hammer the sample surface. NanoScript^TM^ macro Litho functions were listed in the Nanolithography Software 5.12 [21], provided by Veeco Instruments Inc.

The *scan size* command defines the allowable dimensions for the lithographic image. For complete control of lithography functions, the physical limits must be carefully predefined so that the executive commands do not exceed those limits.

Generally speaking, the choice of the probe is dependent on the sample and the AFM technique. For this study, an ordinary, commercially available silicon probe NSG-10 was selected for hammering patterns on structure-tailored thin SEBS film. During the nanolithography process, AFM is operated on tapping mode, and the cantilever, driven by a dither piezo actuator, vibrates near its resonance frequency. The vibration amplitude is kept constant by a feedback loop that changes the distance between the sample surface and the cantilever. Changes in the *Z*-piezo extension are used to reconstruct the topography of the sample. In order to create patterns on the sample surface, the modulation amplitude given to the dither piezo suddenly increases the value of *V*_reading_ to the value of *V*_writing_. With the oscillation amplitude suddenly increased, the deformation of the thin SEBS film is achieved by increasing the tapping load on the tip. Under the condition of fixed free amplitude (*A*_0_), setpoint amplitude (*A*) was reduced; amplitude ratio (*A*/*A*_0_) was also reduced to less than 0.6, so as to decrease the average tip-to-surface separation [3] and to increase the maximum force exerted on the sample when the tip hammers with larger force, elastic, and plastic deformations occur on the SEBS film surface and lithography patterns can be achieved. In this hammering experiment, the empirical value of setpoint was set at 0.9 for generating the nano-patterns. Meanwhile, in order to avoid as much further indentation to the sample surface as possible, the amplitude ratio (*A*/*A*_0_) is increased to a value above 0.9 to reduce the tapping force on the sample, and the cantilever tip will gently tap the sample surface for lithographic patterns scanning. All images scanning for thin SEBS films were performed using the AFM tip in light tapping conditions (*A*/*A*_0_ =0.9), and both height and phase images [4] were recorded simultaneously.

**Figure 3:**
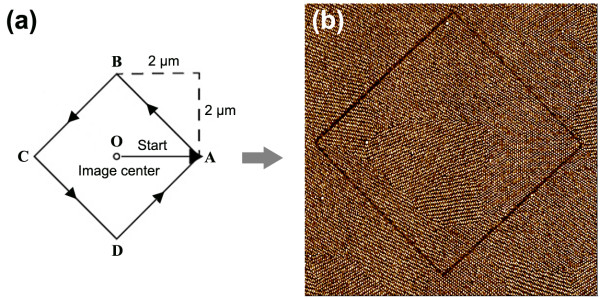
**Designing and fabricating pattern through automatic hammering technique.** (**a**) design program to control the tip-moving route path and (**b**) phase image of the diamond pattern (image size: 4.5 × 4.5 μm^2^ ).

Based on the software and the fabricating method, automatic hammering nanolithography was put forward, and an example for generating diamond was represented in the following. The diamond pattern was designed in Figure 3a; according to the matching program, the tip hammered the film surface after moving 2 μm from the center of the scan field to create a diamond with a diagonal scale of 4 μm. The matching C program was listed as the following:

#include < litho.h>

extern “C” __declspec(dllexport) int macroMain()

{

LITHO_BEGIN

LithoDisplayStatusBox(); // displays litho status box

LithoScan(false); // turns off scanning

LithoCenterXY(); // moves tip to the center of the field

// declares cyclic variable

int i = 1;

double size = 2.0; //declares translation distances of 2 μm from *O* to *A*

double rate = 1.0; // declares the rate of 1 μm/s for moving tip

double vSetPoint = 0.90; // declares a Setpoint of 0.9 for when hammering surface

LithoTranslate( size, 0, rate); // moves the tip to the first corner (*A*)

LithoSet(lsSetpoint,vSetPoint); // sets the Setpoint value at 0.9

for(i = 1;i < =5;i++) // hammers the same patterns repeatedly for five times

{//the four sides are scribed using size and rate in the positive and negative directions

LithoTranslate(−size, size, rate);

LithoTranslate(−size, −size, rate);

LithoTranslate( size, −size, rate);

LithoTranslate( size, size, rate);

}

LITHO_END // ends the lithography program

return 0; } // this command returns the microscope to normal scanning

**Figure 4:**
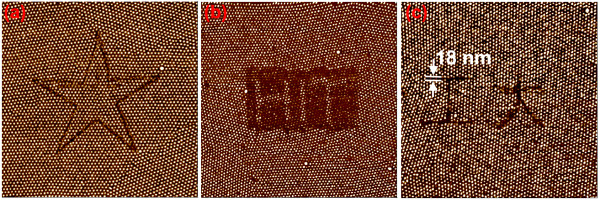
**AFM phase images of nano-patterns by automatic hammering technique.** (**a**) imprinted 5-pointed star pattern; (**b**) embossed pattern-word HIT; (**c**) imprinted Chinese characters. Image size: 2.0 × 2.0 μm^2^.

The command sentence of Lithoset*(*lsSetpoint*,* vSetPoint*)* was used to set the tapping load for the AFM tip, and the sentence double vSetPoint = 0.90 was used to modulate the *setpoint* value at 0.9 for hammering nano-patterns. As shown in Figure 3a, the LithoTranslate (size, 0, rate) command was used to move the tip from center *O* to point *A* by a distance of 2 μm; the command of LithoTranslate (−size, size, rate) moved the tip from point *A* to *B* to hammer side *AB* of the diamond by declaring the parameters − size, size or movements in negative *X* and positive *Y*, and the moving rate was 1.0 μm/s by declaring the sentence of double rate = 1.0. The tip then proceeded to hammer the remaining sides of the diamond shape (sides BC, CD and DA) onto the sample surface. A loop program for (i = 1;i < =5;i++) was written into the program so that the tip continued to hammer the pattern route continuously for five times to improve surface indentation and resolution, creating much clearer patterns. After the AFM tip completed all the sentences or commands in the above program, a perfect diamond pattern on the structure-tailored thin SEBS film was generated automatically, as shown in Figure 3b. Besides the simple patterns, more complicated imprinted or embossed high resolution nano-patterns can also be generated by using the automatic hammering method, simply by designing and changing the defined corresponding program that controls the tip movement route. Figure 4a-c shows a variety of more complicated patterns; Figure 4a shows an imprinted five-pointed star, Figure 4b shows an embossed word HIT, and Figure 4c shows imprinted Chinese characters with minimum linewidth of 18 nm. All the nano-patterns prove that automatic hammering method can be used to fabricate more complicated patterns with high resolution and speed.

**Figure 5:**
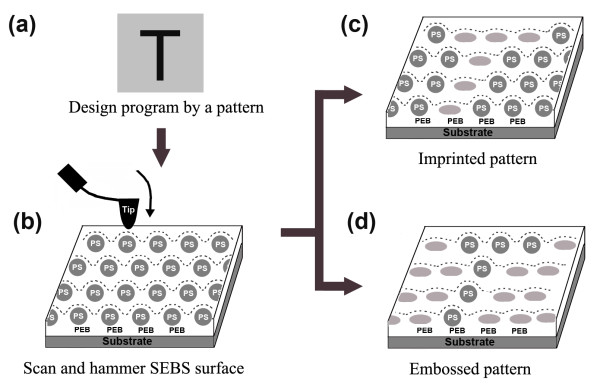
**Schematic of mechanism for hammering imprinted and embossed patterns automatically.** (**a**) design a corresponding program for a planned pattern, i.e., T; (**b**) scan the tailored SEBS film in which hexagonal-spherical PS microdomains are embedded in PEB matrix. The curved dash lines over PS spheres is an AFM tip indented surface under light tapping conditions (non-lithographed SEBS surface); (**c**) imprinted and (**d**) embossed T patterns generated by the auto-hammering the tailored SEBS film, where the light colored PS ellipsoids with dashed lines are the selectively deformed PS spherical microdomains under hard hammering force designed by the program.

A schematic process for the automatic hammering lithography is shown in Figure 5a-d. First and foremost, a computer program is of upmost importance for a perfect outcome, so a corresponding program is designed for ‘writing’ a pattern, i.e., ‘T’, represented in Figure 5a. The next step involves scanning over the SEBS film surface to confirm the suitability for AFM tip hammering, and the sample surface is scanned in light tapping mode to select a large area suitable for hammering. Shown in Figure 5b is the hexagonal-spherical PS microdomains embedded in the PEB matrix. The curved dash lines over PS spheres represent an AFM tip indented surface under light tapping conditions (non-lithographed SEBS surface); Lastly, the sample surface was hammered by oscillating AFM tip with a predefined route and load by the program. The plastic PS microdomains can therefore be selectively hammered from spheres to ellipsoids, and the indentation contrast between the spherical and ellipsoidal PS microdomains gives rise to either imprinted T pattern (Figure 5c) or embossed T pattern (Figure 5d).

In the past studies of mechanical force nanolithography [15-17,22,23], tip plowing causes a significant reduction in the practical linewidth resolution, with cantilever torsion producing edge irregularities or sidewalls of piled up debris on each side of a scratch line. The edge irregularities can be overcome on SEBS films with hexagonal-spherical microstructure by using ATHN manual procedures [19]; while the hammering process and overall operation are very difficult, being limited to hammering individual lines in any single step of the procedure. This costs much time, and it is impossible to hammer out duplicate identical patterns forever. However, the improved automatic method for hammering nanolithography solves these mentioned problems, which saves much time in fabricating complex patterns. A complicated pattern can be completed in less than ten minutes depending on the predesigned program, and the linewidth precision and the success rate can also be significantly improved. In addition, the improved automatic method has a good reproducibility and repeatability; the corresponding design program can be hammered repeatedly to duplicate the same patterns for many times, and the pattern size can also be changed easily through adjusting the program parameters. This new automatic hammering method overcomes some common drawbacks of previous AFM nanolithography methods [15-17,22,23], such as expertise operating skill, expensive diamond tips, tip wearing, and low efficiency. Most importantly, it is well known that the existence (or absence) of a spot of the local indentation can represent ‘0’ or ‘1’ of a digital mode in a data storage context. The preexisted dot array pattern and shape memory effect of this block copolymer media therefore offer particular applicability in the field of high density data storage should we are able to selectively indent dot-array patterns at predefined positions using this newly developed auto-hammering method, which will be reported later. The limitation of the present hammering nanolithography is that the writing media has to be the specially tailored block copolymer films.

## Conclusions

A high-speed automatic nanolithography technique is proposed in this article, in which a vibrating AFM tip is used to generate both imprinted and embossed nano-patterns with 18 nm linewidth resolution on structure-tailored thin SEBS block copolymer film. In the process for generating the complex nano-patterns, the route path of the tip movement is precisely controlled in accordance to a predefined computer program. Results show that this technique allows any complex high resolution nano-patterns to be hammered on thin SEBS film of spherical microstructure using conventional AFM instrumentation in tapping mode, ordinary silicon tip, and appropriately designed pattern program. In comparison with conventional mechanical force nanolithography, this automatic nanolithography technique has some advantages, such as having no edge irregularities or sidewalls around the imprint pattern and having high resolution and high efficiency. Therefore, the auto-hammering method will become a promising lithography technique in the near future and has the potential for application in the fields of data storages, microelectronics, nanotechnology, and miniature sensors.

## Competing interests

The authors declare that they have no competing interests.

## Authors’ contributions

XH carried out the operation of automatic hammering, participated in the sequence alignment, and drafted the manuscript. YY participated in designing the programs for fabricating patterns. YW conceived of the study, participated in its design, and performed the statistical analysis. All authors read and approved the final manuscript.
